# Splinting of Longitudinal Fracture: An Innovative Approach

**DOI:** 10.1155/2016/5083874

**Published:** 2016-05-09

**Authors:** Rashmi Bansal, Priyanka Chowdhary, Anuraag Gurtu, Nakul Mehrotra, Abhinav Kishore

**Affiliations:** Department of Conservative Dentistry & Endodontics, Institute of Dental Sciences, Bareilly 243006, India

## Abstract

Trauma may result in craze lines on the enamel surface, one or more fractured cusps of posterior teeth, cracked tooth syndrome, splitting of posterior teeth, and vertical fracture of root. Out of these, management of some fractures is of great challenge and such teeth are generally recommended for extraction. Literature search reveals attempts to manage such fractures by full cast crown, orthodontic wires, and so forth, in which consideration was given to extracoronal splinting only. However, due to advancement in materials and technologies, intracoronal splinting can be achieved as well. In this case report, longitudinal fractures in tooth #27, tooth #37, and tooth #46 had occurred. In #27, fracture line was running mesiodistally involving the pulpal floor resulting in a split tooth. In teeth 37 and 46, fractures of the mesiobuccal cusp and mesiolingual cusp were observed, respectively. They were restored with cast gold inlay and full cast crown, respectively. Longitudinal fracture of 27 was treated with an innovative approach using intracanal reinforced composite with Ribbond, external reinforcement with an orthodontic band, and full cast metal crown to splint the split tooth.

## 1. Introduction

Conservation is becoming the basis of dentistry due to advancements in adhesive materials and technologies [1]. Longitudinal tooth fractures, which were once considered irreparable, can now be preserved [2]. The success of such teeth depends on its early identification and management. Based on location, direction, extent, prognosis, and treatment modalities, they can be classified as craze lines on the enamel surface, one or more fractured cusps of posterior teeth, cracked tooth syndrome, splitting of posterior tooth, and vertical fracture of root [3].

Craze lines are the least complicated type of fractures, extending on any surface buccally or lingually running through the marginal ridges. Fractured cusp initiating from the crown may extend subgingivally, through the buccal or lingual groove. Split tooth type of fractures has subgingival extension involving both the marginal ridges. The more centered the split occlusally, the greater the tendency to extend apically [2].

**Figure 1:**
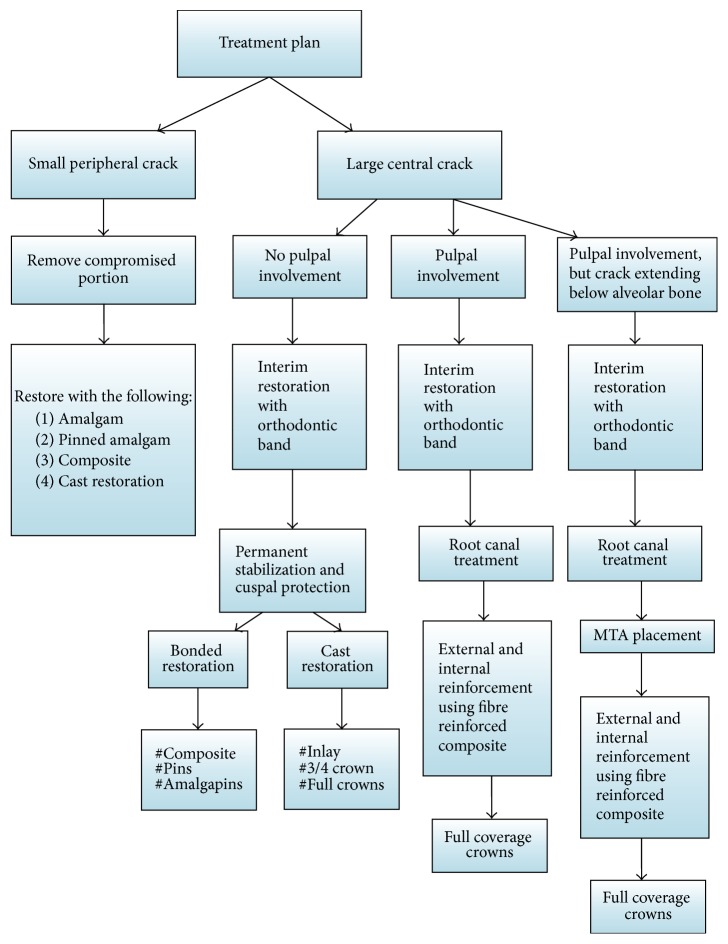
Treatment protocol for longitudinal fracture of teeth.

Posterior tooth fractures occur mainly due to masticatory forces, large restoration, or endodontically treated teeth and rarely due to acute trauma [4, 5]. Management of different longitudinal fractures depends upon the extent of fracture line and vitality of the tooth (Figure 1). Amongst various types of longitudinal fractures, the most devastating and controversial to treat is the split tooth involving the pulp chamber. A literature search for the management of split tooth reveals that if the fracture extends to a root surface, the mobile segment can be removed followed by surgical crown lengthening and orthodontic extrusion of the retained segment [2]. The rationale behind such treatment is that when fracture line extends below the attachment it becomes a site for infection, subsequently leading to bony defect. If the fractured segment of the split tooth is immobile, in such situation the cusp removal is not required; instead a full coverage restoration can be placed to hold the segments. For reinforcement-ligature wires [6], composite resin [7], adhesives [8], full coverage crowns, intentional reimplantation [9], and lasers like CO_2_ and Nd-YAG, used to fuse the fracture segments [10, 11], have been successfully tried. In all these cases, the fracture line involving the pulpal floor was sealed with adhesive restorations, which is not a bioactive material and cannot promote cementogenesis at fracture site in the furcation area.

This paper presents an innovative approach to the management of split tooth (tooth #27) due to trauma by internal and external reinforcement. Cementogenesis was promoted at the fracture site in the furcation area by using MTA as a bioactive material. Further, in the same patient, fractured cusps of teeth 37 and 46 were also successfully managed by cast gold inlay and full cast crown, respectively.

## 2. Case Presentation

**Figure 2:**
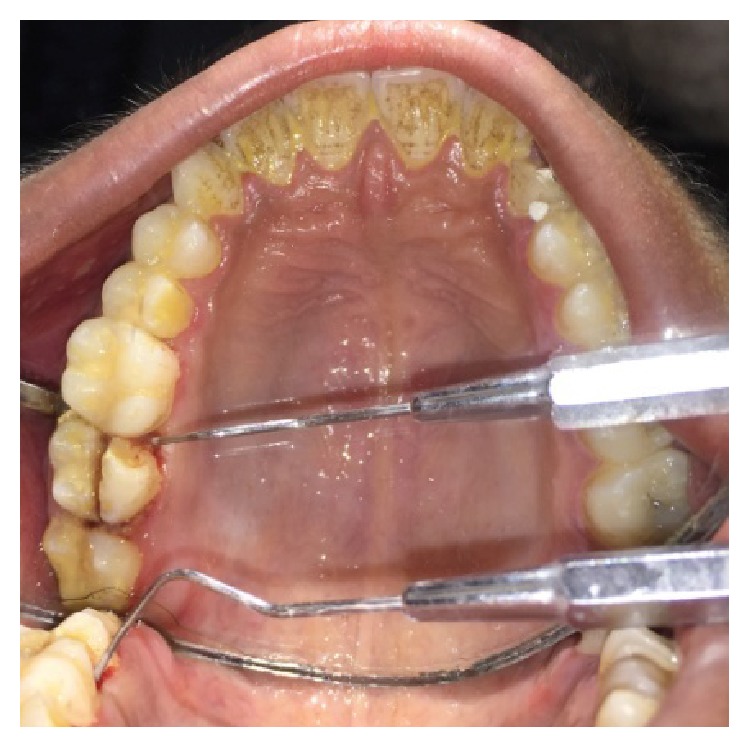
Preoperative photograph showing split tooth of 27.

**Figure 3 fig3:**
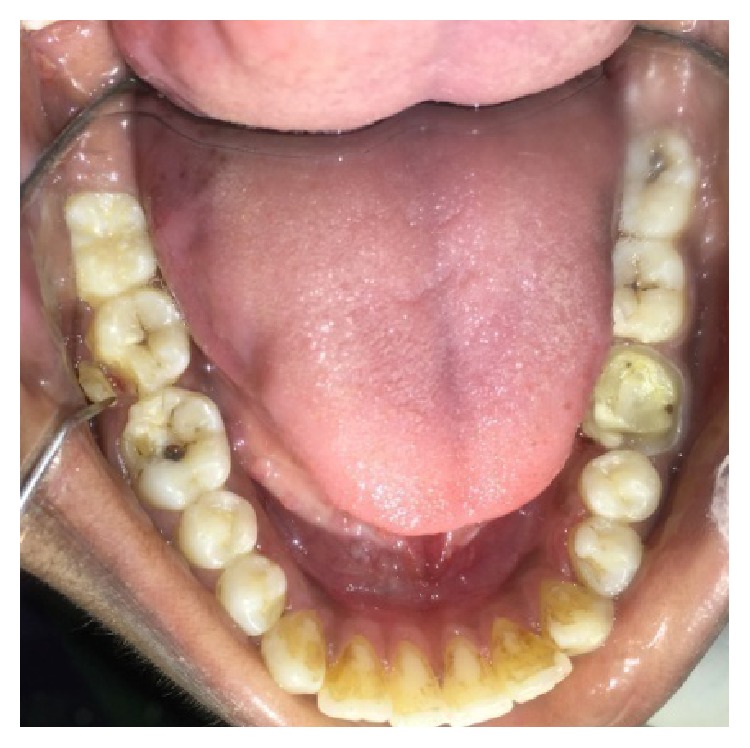
Preoperative photograph showing fractured cusps in 37 and 46.

**Figure 4 fig4:**
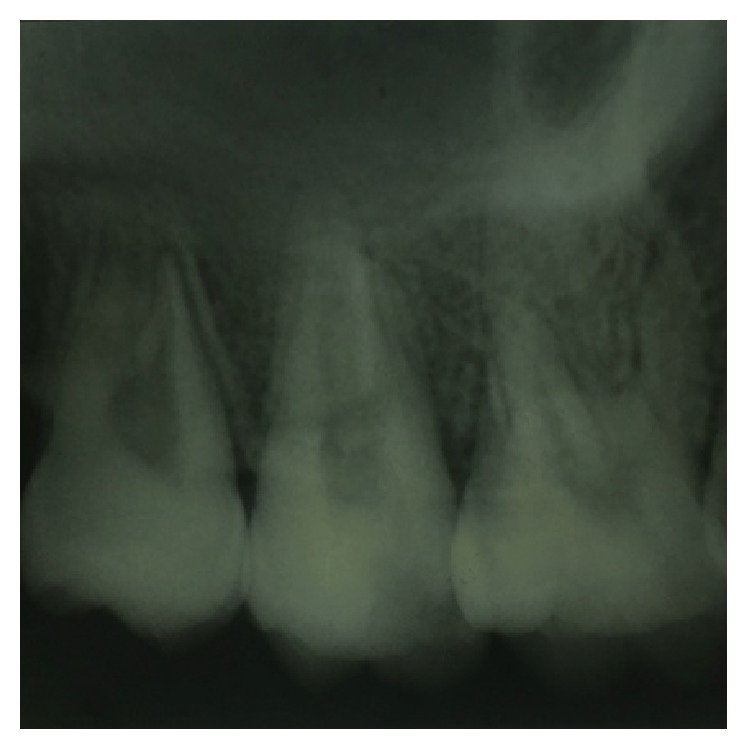
Preoperative radiograph of 27.

**Figure 5 fig5:**
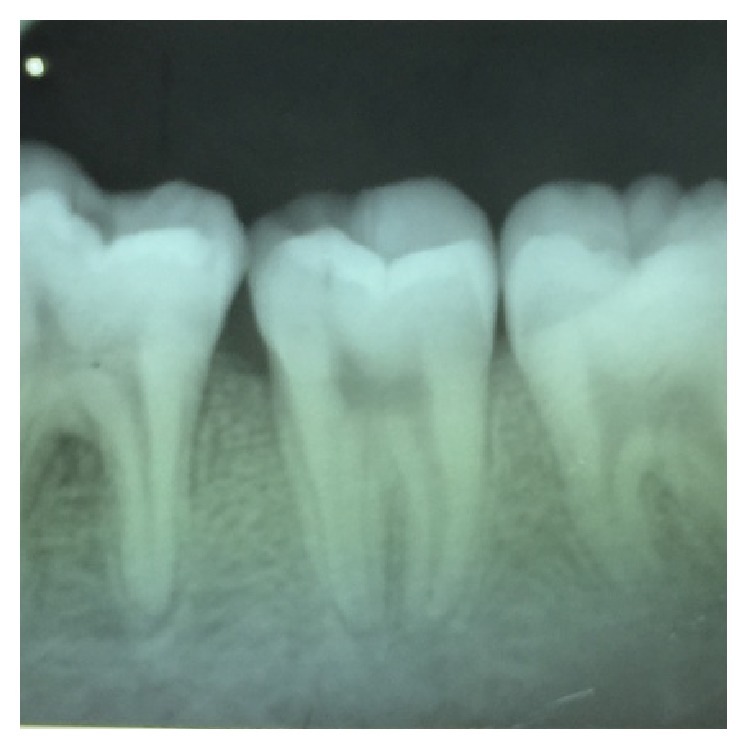
Preoperative radiograph of 37.

**Figure 6 fig6:**
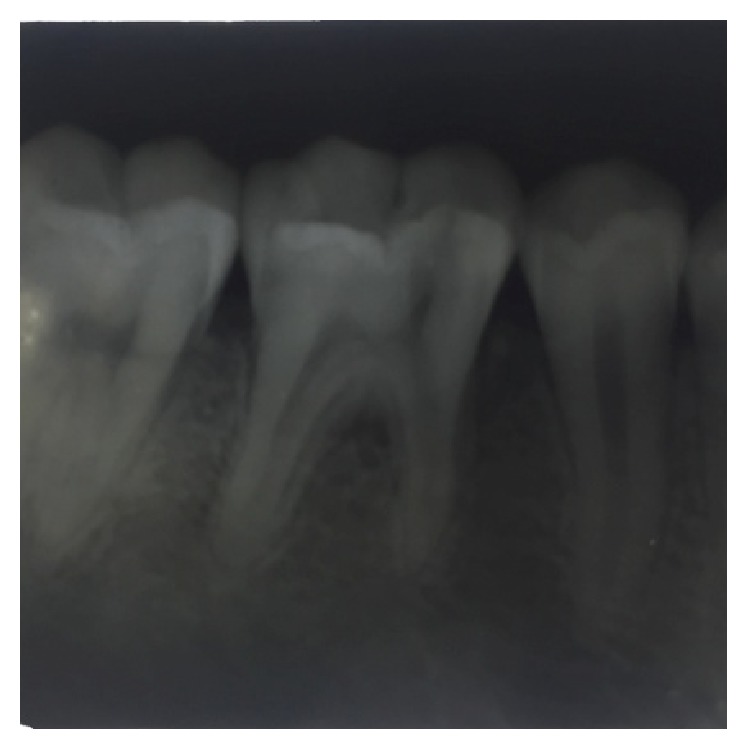
Preoperative radiograph of 46.

**Figure 7 fig7:**
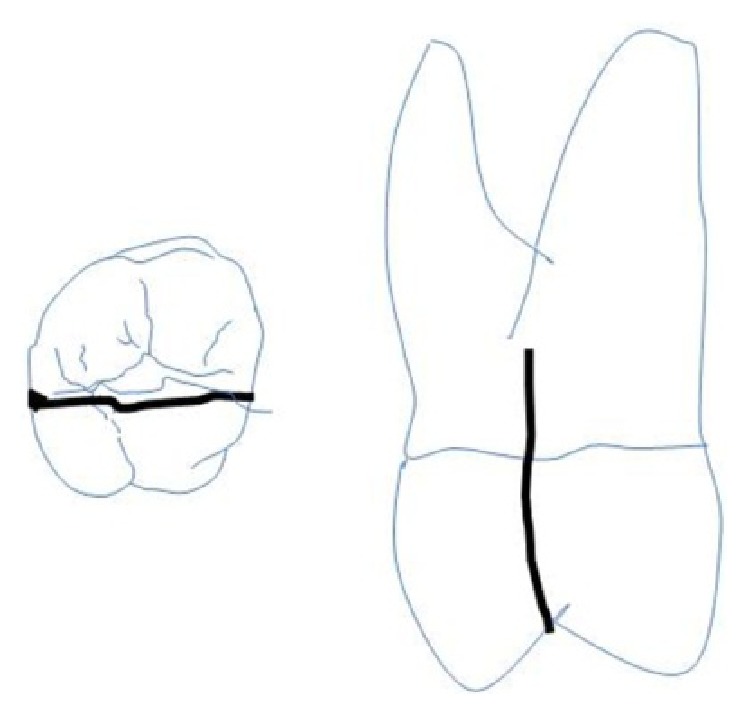
Extent of fracture line in 27.

**Figure 8 fig8:**
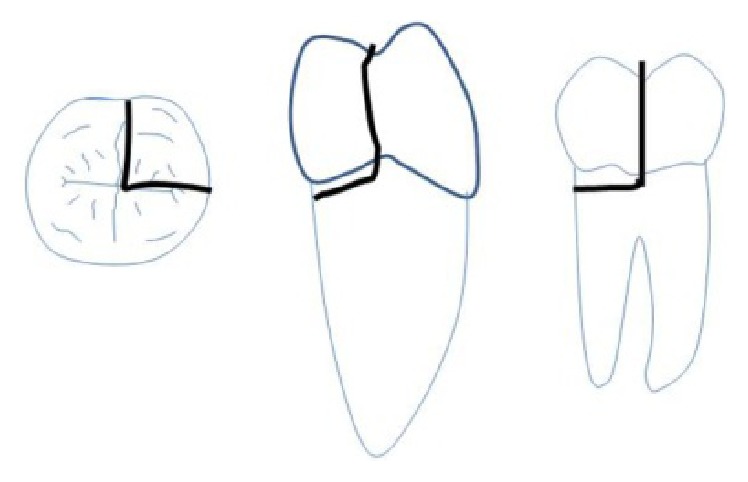
Extent of fracture line in 37.

**Figure 9:**
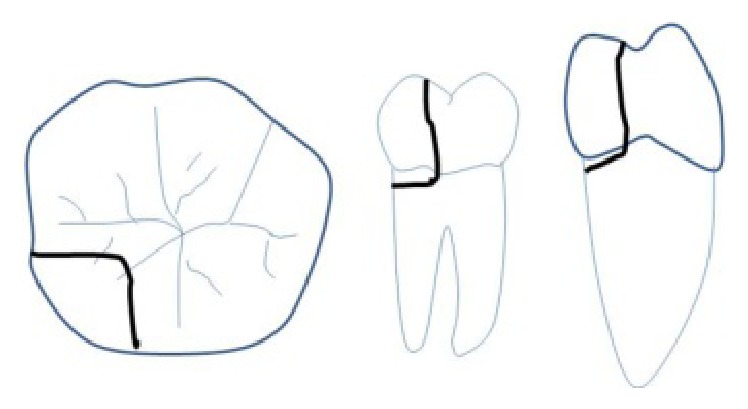
Extent of fracture line in 46.

A male patient, 19 years of age, came to the Department of Conservative Dentistry and Endodontics with chief complaint of multiple fractured teeth in both upper and lower jaws causing pain and discomfort for the last two weeks. The patient gave a history of trauma two weeks ago due to injury by hand pump handle. Extraoral examination revealed no significant findings. Intraoral examination of all quadrants revealed longitudinal fractures involving 27, 37, and 46 (Figures 2 [Fig fig3] [Fig fig4] [Fig fig5] [Fig fig6] [Fig fig7] [Fig fig8]–9).

On visual inspection of 27, the fracture line was running mesiodistally involving both the marginal ridges (Figures 2 and 7). The fracture segments cannot be displaced, but they can be visualized by wedging with a probe (Figure 2). There was no dentine between the fractured segments on the pulpal floor. The fracture line had extended subgingivally through the pulp chamber into the furcation area. Exploration of periodontium along the fracture line indicates no sign of periodontal attachment loss, although tooth was tender on percussion and nonvital on pulp testing. An intraoral periapical radiograph revealed that lamina dura of mesiobuccal and distobuccal root was involved. Fracture line was not appreciable on the radiograph (Figure 4). It was diagnosed as a split tooth of 27 involving the pulpal floor.

On examination of the lower left quadrant, there was a fracture of a mesiobuccal cusp with respect to 37. The fracture line had both mesiodistal and buccolingual components. It was running cervically from buccal groove towards the centre of occlusal surface and then extending mesially involving the mesial marginal ridge (Figure 8). Both fractured segments were in position but can be visualized by exploring with a probe (Figure 3). Further, the subgingival extent of fracture line was 1 mm below the cementoenamel junction (CEJ), and it was not involving the pulp chamber. Periodontium was intact along the fracture line. An intraoral periapical radiograph revealed intact lamina dura (Figure 5). The tooth was not mobile and not tender on percussion. Vitality test was positive. It was diagnosed as fractured mesiobuccal cusp with respect to 37, not involving the pulp.

On examination of the lower right quadrant, there was a fracture of mesiolingual cusp with respect to 46. The fractured segments were not displaced but could be separated by exploration with the probe. Further, the fracture line was extending up to the coronal third of the root surface, without involving the pulpal floor (Figure 9). Periodontium was not compromised along the fracture line and percussion test was negative. Vitality test was positive. Intraoral periapical radiograph revealed fracture line (Figure 6). It was diagnosed as fractured mesiolingual cusp with respect to 46.

Comprehensive treatment was planned for each tooth depending on the vitality and extent of fracture line mesiodistally, buccolingually, and gingivally. Before initiation of the treatment, diagnosis, treatment, and prognosis were discussed with the patient.

**Figure 10:**
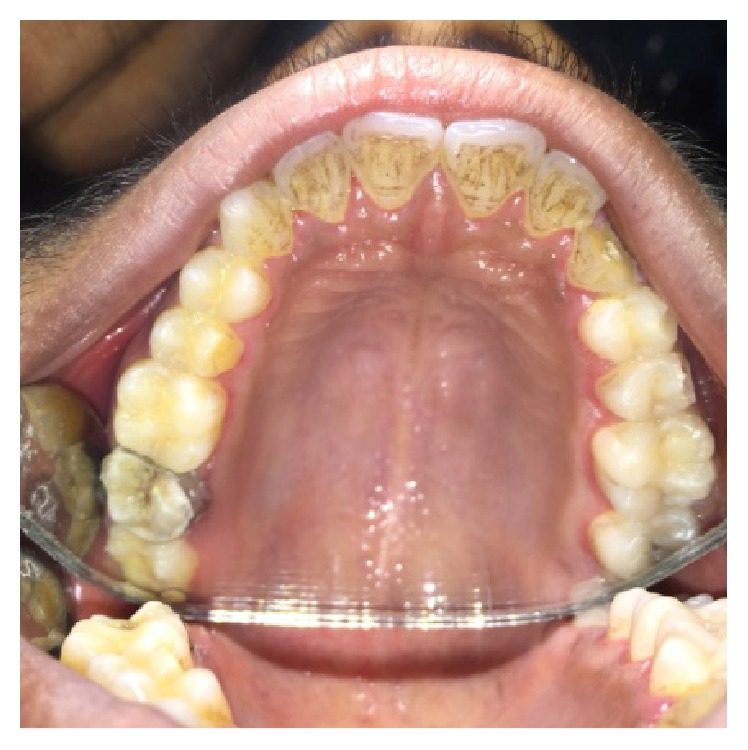
Photograph showing stabilization of split tooth with respect to 27 using orthodontic band.

**Figure 11:**
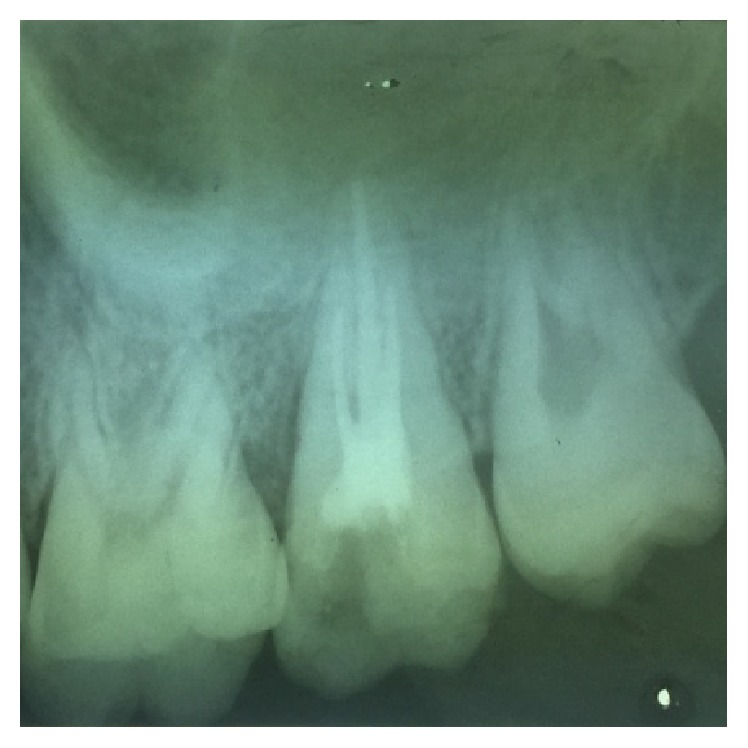
Postobturation radiograph with respect to 27.

**Figure 12:**
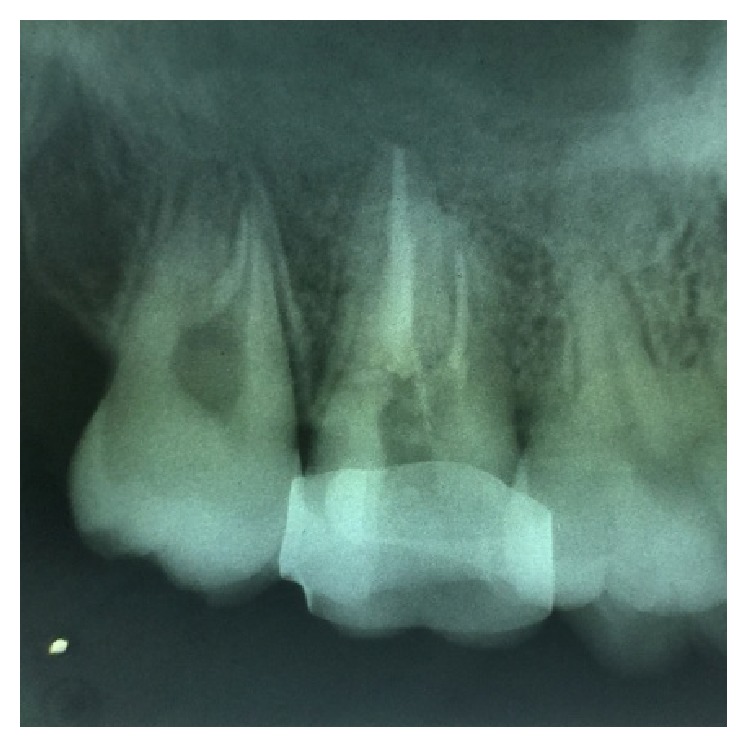
Radiograph showing 3 mm of intracanal space preparation with respect to 27.

**Figure 13:**
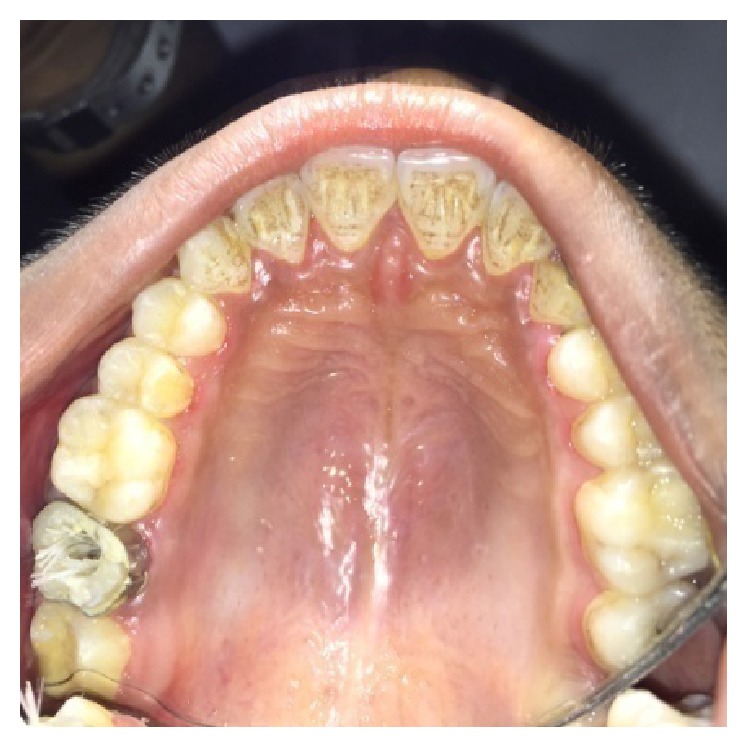
Photograph showing 3 mm placement of Ribbond in the canal.

**Figure 14:**
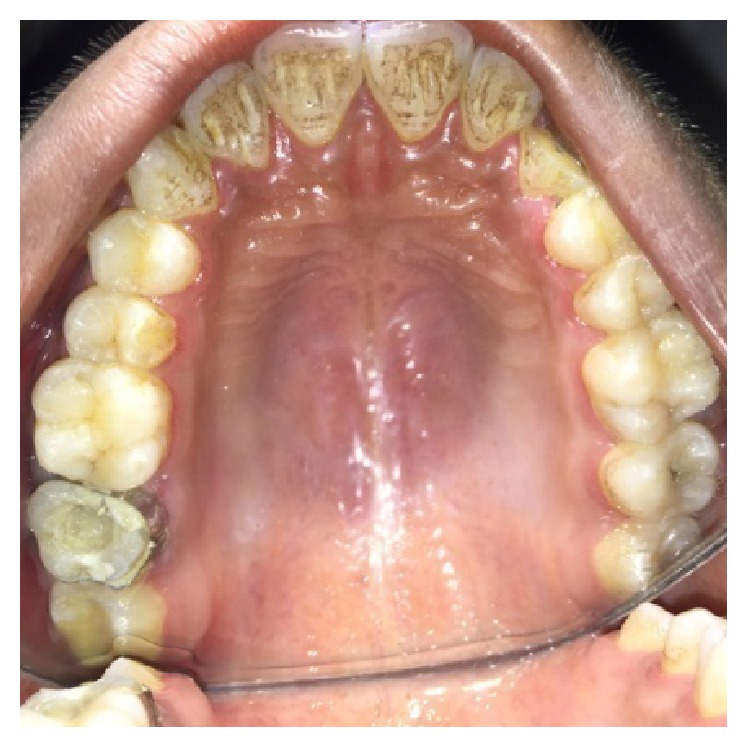
Photograph showing meshwork of fibre in the pulp chamber.

**Figure 15:**
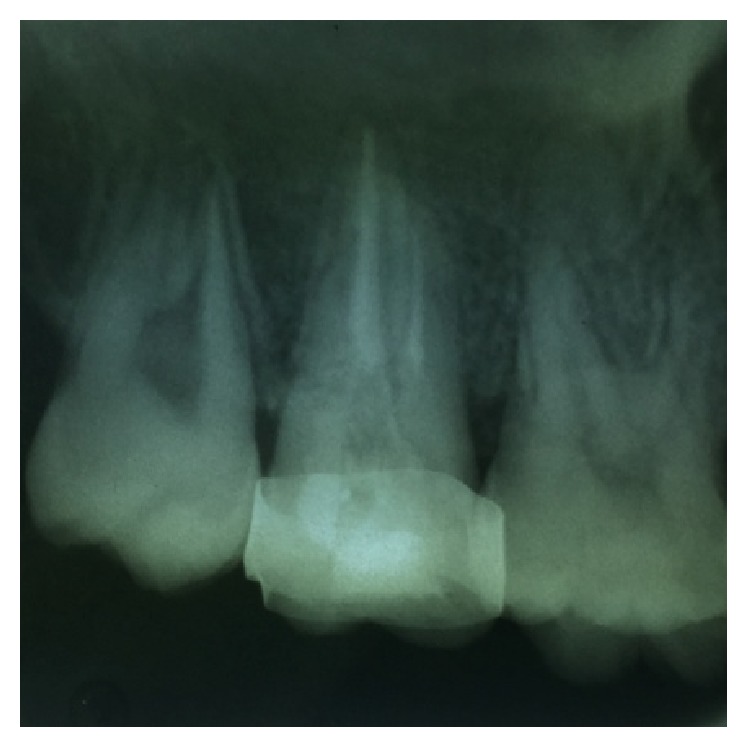
Radiograph showing internal reinforcement with Ribbond in 27.

**Figure 16:**
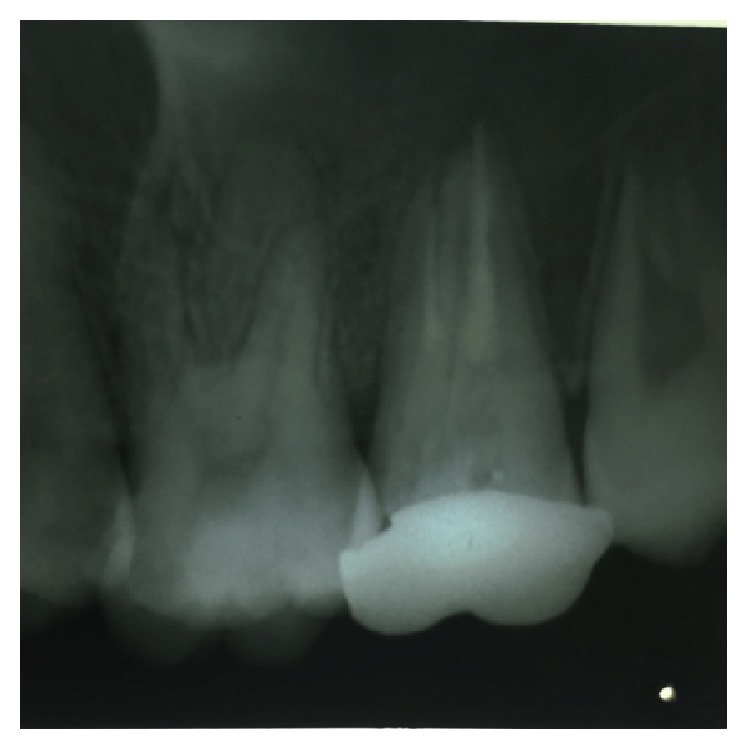
Radiograph showing external reinforcement with metal crown in tooth 27.

Immediately after the diagnosis, the tooth was stabilized using orthodontic band (RMO 0.180 × 0.005 mm) (Figure 10). In the same appointment, endodontic treatment was initiated. Fracture line was cleaned of any debris with 30% H_2_O_2_ (Shree Sai Chemicals, Ghaziabad, Uttar Pradesh, India). The biomechanical preparation was done using Rotary V-Taper (30/0.04%) (SS White) (Figure 11). Single cone gutta-percha obturation using MTA Fillapex, as the root canal sealer, was completed. Mineral Trioxide Aggregate (ProRoot MTA, Dentsply, DeTrey, Germany) was placed on the pulpal floor at the fracture site where dentine was missing. A wet cotton pellet was placed in the pulp chamber followed by temporary restoration. The patient was recalled after 3 days. In the next appointment, temporary restoration was removed and access cavity was thoroughly debrided with saline and dried. From each canal, 3 mm of gutta-percha was removed with Peeso Reamer size-3 (Dentsply Maillefer, Ballaigues, Switzerland), and preparation was checked radiographically (Figure 12). Three pieces of 1 cm Ribbond (Ribbond Inc., USA) fibre were taken. The root canal orifices were etched and rinsed and bonding agent was applied. Into each of the three canals, 3 mm of Ribbond fibres was inserted and was stabilized in the root canal with the help of flowable composite (G-aenial Universal Flo, GC Corporation, Tokyo, Japan) and cured (Figure 13). After curing, around 7 mm of remaining fibres was kept outside the canal orifice. Then, these fibres were spread on the floor of the pulp chamber and intermingled to create a meshwork for the internal reinforcement (Figure 14). A flowable composite was placed over this fibrous meshwork and manipulated slightly with the help of plastic instrument (API) along the buccolingual and mesiodistal directions so as to form a unique composite Ribbond meshwork along the floor of the pulp chamber which was later cured. Subsequently, core buildup composite (Tetric Ceram Ivoclar Vivadent, Germany) was done. After 15 days, the orthodontic band was removed and crown preparation was done. Temporary crown was cemented which was later replaced by metal ceramic crown (Figures 15 and 16).

**Figure 17:**
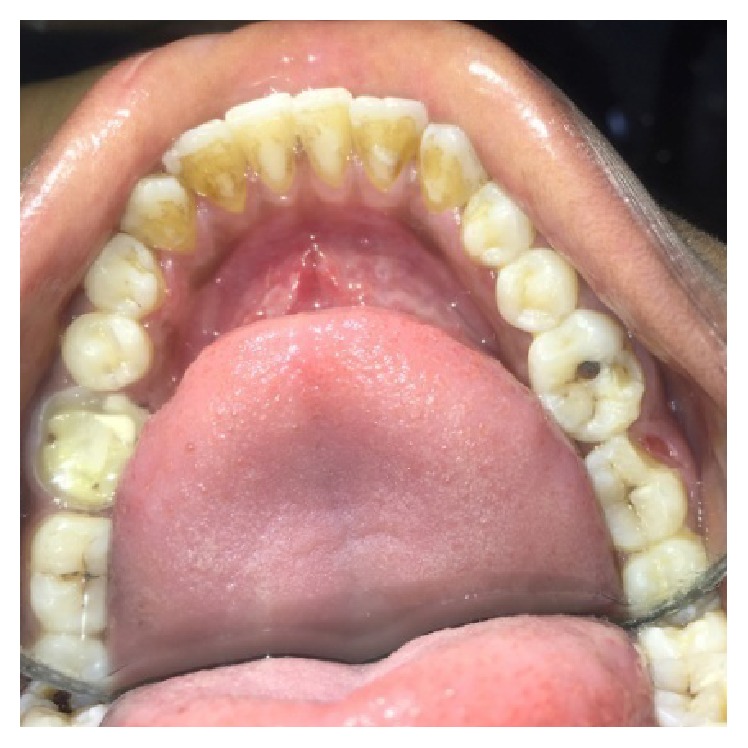
Mesiobuccal cusp in tooth 37 removed.

**Figure 18:**
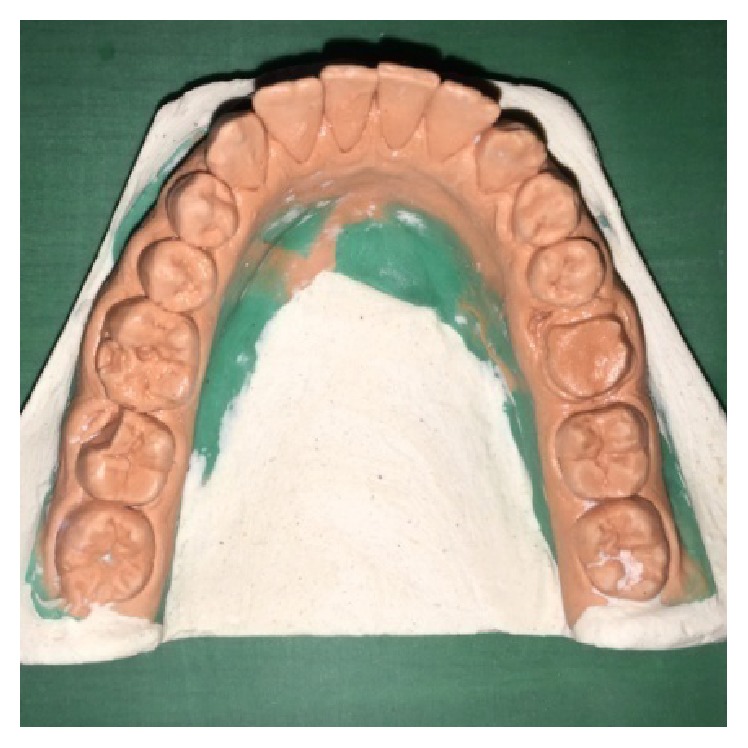
Preoperative cast of tooth 37.

**Figure 19:**
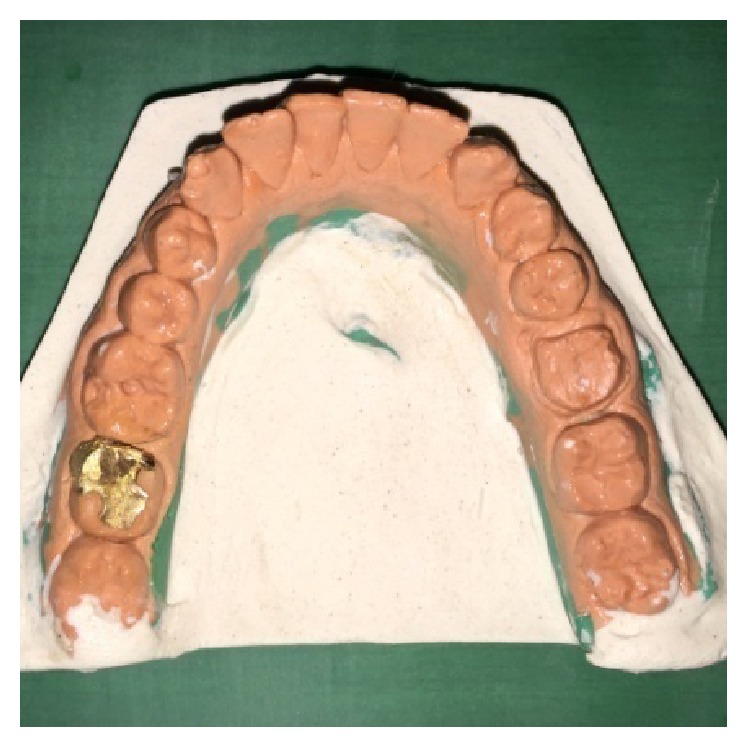
Inlay casting try-in on the cast with respect to 37.

**Figure 20:**
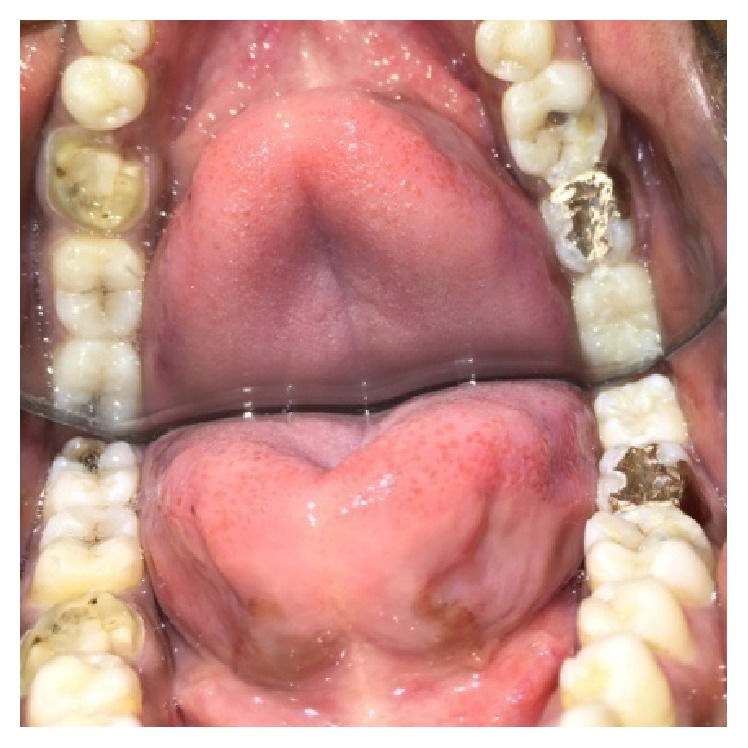
Photograph showing inlay luted with respect to 37 (occlusal view).

**Figure 21:**
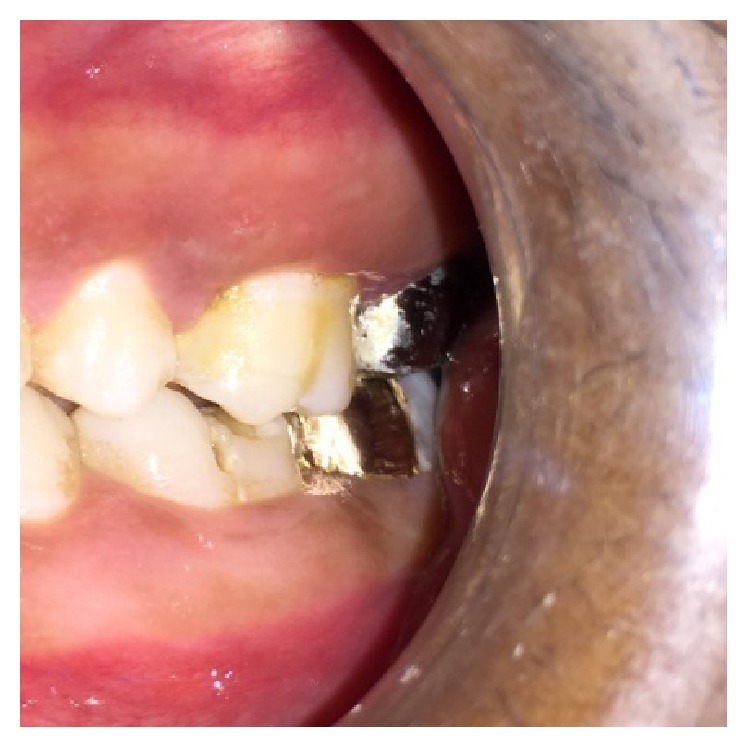
Photograph showing inlay luted with respect to 37 (buccal view).

**Figure 22:**
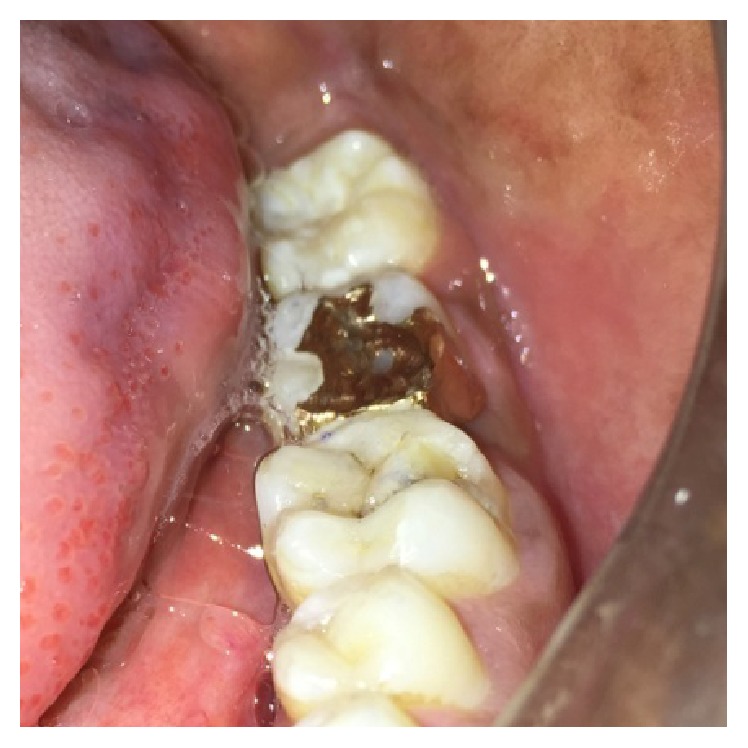
Photograph showing inlay luted with respect to 37 (occlusal view).

**Figure 23:**
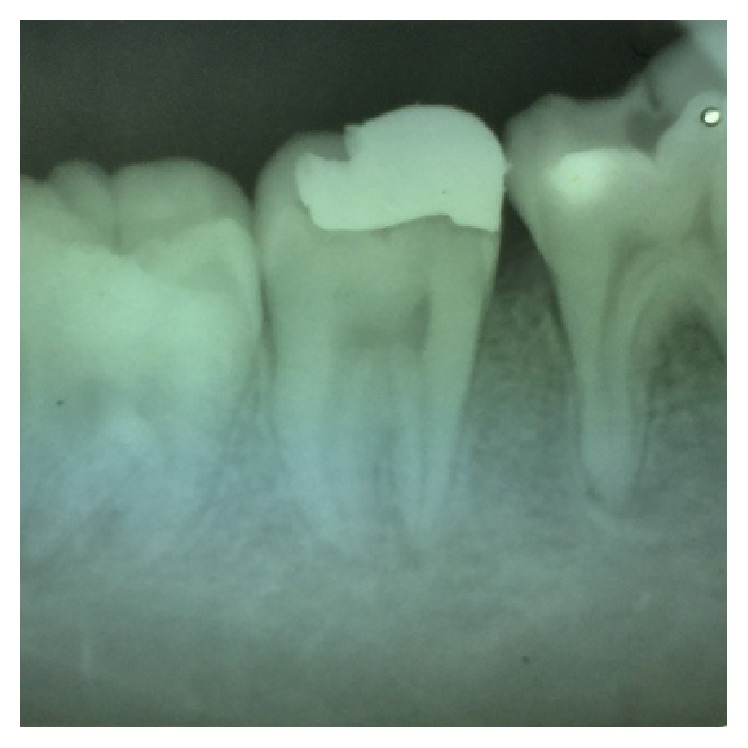
Radiograph showing luted inlay with respect to 37.

Fractured mesiobuccal cusp in 37 was removed carefully (Figure 17) and tooth preparation was done for class II cast metal inlay with cusp capping. Tooth preparation was extended more apically, and an indirect pattern was fabricated (Figures 18 and 19). Cast gold inlay was fabricated, luted using glass ionomer cement type I (GC Corporation, Tokyo, Japan) (Figures 20, 21, and 22), and checked radiographically (Figure 23).

**Figure 24:**
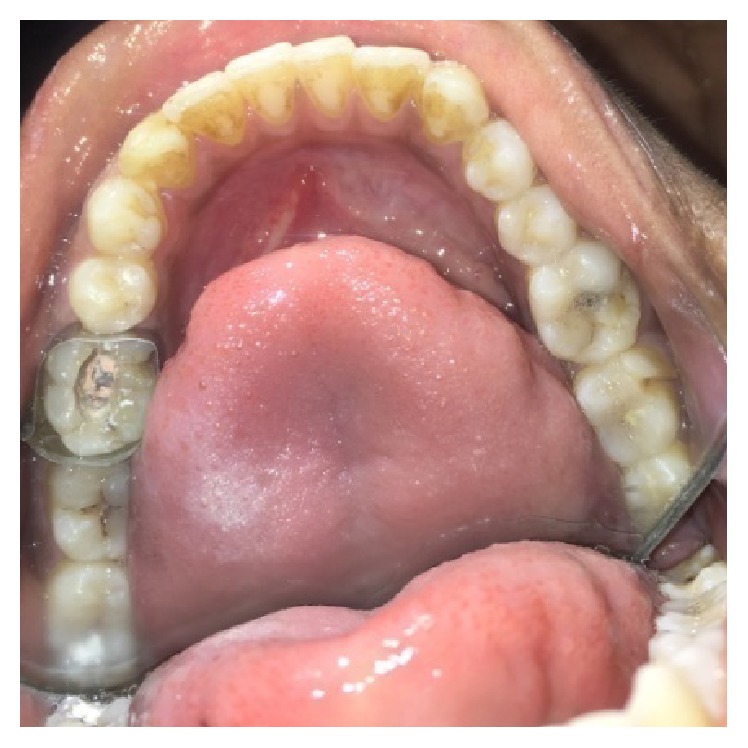
Photograph showing stabilization of fractured cusp with respect to 46.

**Figure 25:**
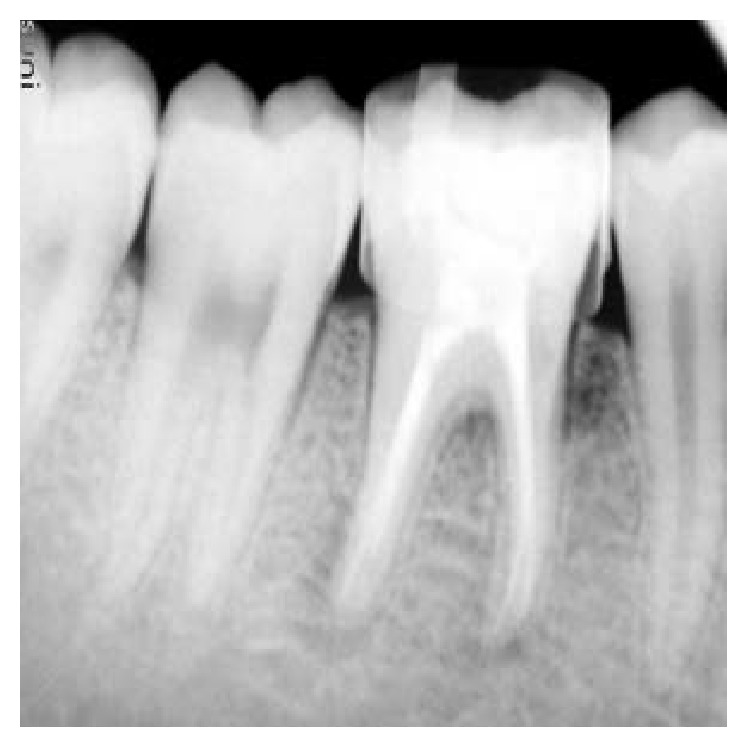
Postobturation radiograph of 46.

As the fracture line was not involving the pulp chamber in 46 but was extending subgingivally, and tooth was vital initially, the fractured cusp was not removed; instead it was bonded with the help of composite resin and stabilized with orthodontic band (Figure 24). On subsequent appointment, the tooth had become nonvital and a sinus tract had also appeared in its relation. Hence, endodontic treatment was completed (Figure 25). Internal and external reinforcement were achieved in the same manner as mentioned for tooth 27.

## 3. Discussion

Two classic patterns of longitudinal fracture were seen in this case report. In one (#27), the fracture was located centrally, extending to the pulp, and, in others (#37 and #46), the fracture was more peripherally directed and resulting in a cuspal fracture.

In tooth #27, the fracture was crossing both the marginal ridges involving the pulpal floor and there was no dentine connection between the two segments. Such teeth are usually difficult to restore and generally advised for extraction, as granulation tissue grows in the furcation area which results in loss of periodontal attachment. Further, bonding of two fragments together is difficult as the cuspal flexure during occlusion may result in failure of the bond [12]. However, with advancements in biomaterials and adhesive materials both aspects were successfully managed in tooth #27. MTA being bioactive, biocompatible, and having biomineralizing properties allows cementum to grow at the furcation area and aids in the regeneration of periodontal ligament [13]. Hence, MTA was used to seal the pulpal floor at the fracture site. Perfect approximation of split section was achieved with orthodontic banding. As MTA sets in the presence of moisture [14, 15], matrix was not required to contain the MTA. Since MTA is weak in compressive strength, it was reinforced by flowable composite to bear the occlusal forces.

The tooth was further reinforced internally and externally using orthodontic band and fibre Ribbond. The orthodontic band provided a good interim stabilization of fracture segment. Recent studies have proved that orthodontic band reduces cuspal flexure by one-half compared to teeth without bands [16]. Internal reinforcement was achieved using Ribbond, possessing high strength fibres with cross-link weave. Ribbond also has enhanced bond ability, thereby resisting crack opening and creating a strong bond between the fractured segments. Stresses can be redistributed along the direction of the fibre to intact portions of the teeth and away from the bonded surface [17].

To reduce the polymerization shrinkage, incremental placement of composite using pulse curing technique was used. This reduced the stress development at the cavosurface margin and reinforced the remaining tooth structure [18]. Full coverage metal crown was fabricated and cemented over the treated tooth, which gave external reinforcement of fractured segments. Literature search reveals case reports on managing such fracture by intentional reimplantation procedure. 4-META adhesive was used to bond split tooth after extraction. With such technique, resorption of root after reimplantation remains a concern. Further intentional reimplantation is an invasive procedure [10]. CO_2_ and Nd-YAG lasers were found ineffective in fusing the segments [11].

In tooth #37, the fracture line was not extending below the cervical line and was not involving the pulp. Cast gold inlay with cusp capping of mesiobuccal cusp was planned instead of composite restoration, as resin shows polymerization shrinkage, which can further displace and weaken the fractured segments, rendering them more susceptible to masticatory forces [19]. During tooth preparation, the preparation margins were extended more apically and bevelling the fractured segment to minimize the displacement [12].

Tooth 46 was vital and fracture line was not involving the pulp. Hence, initially, fracture segment was reattached with composite and stabilized with orthodontic bands. The tooth was examined periodically. After two weeks, tooth became nonresponsive to vitality test and an intraoral sinus also developed in its relation. So root canal treatment was initiated and completed. The tooth was restored and permanently stabilized by internal and external reinforcement as done for tooth #27.

During the endodontic procedure in tooth #27 and tooth #46 to avoid excessive wedging forces during lateral condensation which may cause the split to spread, single cone obturation (30/4%) was done after biomechanical preparation with Rotary V-Taper file (SS White, Lakewood, NJ). For irrigation, metronidazole (JB Chemicals, Bharuch, Gujarat) was used. As the fracture line was involving the palatal canal in tooth #27, MTA based sealer (MTA Fillapex, Angelus, Brazil) was used. The post was also avoided for anchorage as they may exert wedging forces, and, instead, Ribbond fibre reinforced resin was used in its place.

## 4. Conclusion 

With recent advancements in adhesives, split tooth resulting in longitudinal fractures that were once considered irreparable and recommended for extraction can now be successfully restored and reinforced both internally and externally. Further, with the introduction of biomaterials fracture lines involving the pulpal floor can be managed by the apposition of new cement at the fracture site. In addition, vital teeth with the longitudinal fracture of the cusp, not involving the pulp, can be conservatively managed by cast gold inlay or full cast crown.
